# Bacterial Communities Associated with *Culex* Mosquito Larvae and Two Emergent Aquatic Plants of Bioremediation Importance

**DOI:** 10.1371/journal.pone.0072522

**Published:** 2013-08-15

**Authors:** Dagne Duguma, Paul Rugman-Jones, Michael G. Kaufman, Michael W. Hall, Josh D. Neufeld, Richard Stouthamer, William E. Walton

**Affiliations:** 1 Department of Entomology, University of California Riverside, California, United States of America; 2 Department of Entomology, Michigan State University, East Lansing, Michigan, United States of America; 3 Department of Biology, University of Waterloo, Waterloo, Ontario, Canada; Swedish University of Agricultural Sciences, Sweden

## Abstract

Microbes are important for mosquito nutrition, growth, reproduction and control. In this study, we examined bacterial communities associated with larval mosquitoes and their habitats. Specifically, we characterized bacterial communities associated with late larval instars of the western encephalitis mosquito ( *Culex* *tarsalis* ), the submerged portions of two emergent macrophytes (California bulrush, *Schoenoplectus* *californicus* and alkali bulrush, *Schoenoplectus* *maritimus* ), and the associated water columns to investigate potential differential use of resources by mosquitoes in different wetland habitats. Using next-generation sequence data from 16S rRNA gene hypervariable regions, the alpha diversity of mosquito gut microbial communities did not differ between pond mesocosms containing distinct monotypic plants. Proteobacteria, dominated by the genus *Thorsellia* (*Enterobacteriaceae*), was the most abundant phylum recovered from *C* *. tarsalis* larvae. Approximately 49% of bacterial OTUs found in larval mosquitoes were identical to OTUs recovered from the water column and submerged portions of the two bulrushes. Plant and water samples were similar to one another, both being dominated by Actinobacteria, Bacteroidetes, *Cyanobacteria* , Proteobacteria and Verrucomicrobia phyla. Overall, the bacterial communities within *C* *. tarsalis* larvae were conserved and did not change across sampling dates and between two distinct plant habitats. Although *Thorsellia* spp. dominated mosquito gut communities, overlap of mosquito gut, plant and water-column OTUs likely reveal the effects of larval feeding. Future research will investigate the role of the key indicator groups of bacteria across the different developmental stages of this mosquito species.

## Introduction

Recent studies have focused on understanding the role of microorganisms in mosquito biology and ecology, with the prospect of designing effective control strategies for species that vector debilitating diseases [1,2,3]. Among these microorganisms, *Bacteria* play an important role, not only as major components of larval diet [4,5], but also in generating volatiles that attract mosquitoes for oviposition [3,6]. *Bacteria* are thought to provide valuable nutrition for the growth of mosquito larvae, but this is likely dependent on the particular species of *Bacteria* present in the larval habitat and ingested by larvae throughout their development [4,7]. A complex microbial consortium is considered fundamental for normal survival and complete development of mosquito larvae to adults [4,5,7].

Larval *Culex* mosquitoes are generally considered filter-feeders and consume *Bacteria* and many other microorganisms in the water column [5]. Studies have shown that the microbial communities isolated from the mid-gut of laboratory-reared fourth instar *Culex* *tarsalis* Coquillett (a vector of western encephalitis and West Nile viruses) using conventional culturing techniques included several species including *Lactobacillus* , *Micrococcus* sp., *Micrococcus* *candidus* , *Saccharomyces* , *Proteus* *rettgeri* , *Geotrichum*, *Pseudomonas* , and other unidentified Gram-negative bacteria [8,9]. Among these, *Micrococcus* sp. (Actinobacteria), *Lactobacillus* (Firmicutes: *Bacilli*) and *Pseudomonas* (*Gammaproteobacteria*) associated most frequently with *C* *. tarsalis* guts [8,9]. Most genera of *Bacteria* found in the gut of larval *C* *. tarsalis* were also found in the adults, with the exception of *Aerobacter* , *Escherichia* , and *Flavobacterium* [8,9]. However, these studies were based on laboratory-reared mosquitoes, and therefore it is difficult to extrapolate the bacterial composition of the gut of this mosquito species in the natural habitat. *Bacteria* found in the water column can be free-living, single cells, but also occur in clumps, and attached to sediment particles or submerged parts of aquatic plants [10]. In our own work, we have often observed the aggregation of *Culex* larvae at the surfaces of aquatic plants and on the sides of mesocosms during active mosquito production seasons. Others have shown biofilms to be important food resources for the larvae of other mosquito genera e.g., *Aedes* [11,12]. However, the extent to which *Culex* species feed on the biofilm attached to these substrates is unknown.

Emergent macrophytes provide attachment sites, carbon and oxygen for microorganisms, and are purposely planted in treatment wetlands to facilitate the remediation of wastewater [13,14]. In addition, macrophytes produce high amounts of organic matter [13,15], thereby increasing wetland food resources for mosquito larvae [16,17]. Large macrophytes (height > 3 m) such as bulrushes, cattail and common reed, are used extensively in constructed treatment wetlands in California and elsewhere in North America. However, dense stands of large emergent macrophytes such as California bulrush ( *Schoenoplectus* *californicus* [C.A. Mey.] Palla) also enhance mosquito oviposition [18] and reduce the effectiveness of mosquito control strategies by forming a physical barrier to “mosquitocides” and providing shelter from predators such as fish [16].

Therefore, macrophytes that interfere less with conventional mosquito control tactics may be preferable to the large macrophytes planted in constructed treatment wetlands. *Schoenoplectus* *maritimus* L. (“alkali bulrush” or “cosmopolitan bulrush”) has a short growth habit (height: 0.5-1.5 m) and, in most habitats, the above-ground biomass dies off annually in winter [19]. *Schoenoplectus* *maritimus* reproduces primarily by a matrix of rhizomes that provides surface area for beneficial *Bacteria* used in wastewater treatment, while removing fecal pathogens such as *Escherichia coli* from the water column [19]. In addition to supporting larger populations of predaceous insects, the annual phenology, morphology and growth patterns of *S* *. maritimus* are predicted to interfere less with integrated mosquito management strategies than do large emergent macrophytes.

Macrophytes interact with aquatic microorganisms and zooplankton in various ways. Some macrophytes release secondary plant compounds, such as phenols and alkaloids, which affect bacterial communities [15,20,21,22,23,24]. These properties are likely to influence the pollutant removal efficiency of treatment wetlands and, more importantly, the growth of mosquitoes and beneficial invertebrates. Some macrophytes are known to have antimicrobial and zooplankton-repellent properties [21]. For instance, root exudates from *Schoenoplectus* *lactularis* , *Phragmites* *communis* and *Juncus* *maritimus* have been reported to alter bacterial composition in wetlands [21]. Furthermore, macrophytes are thought to provide different structural complexes that affect macroinvertebrate composition and abundance [18,23]. Larval *Anopheles* abundance differed among three structurally different emergent macrophytes in northern California [18]. However, many of these studies failed to address the underlying cause of these variations in invertebrate production.

In this study, we addressed the following objectives: 1) characterize bacterial communities within the larvae of the western encephalitis mosquito sampled from semi-natural habitats; 2) characterize the bacterial communities found in the water column from which the mosquitoes were sampled, and, 3) detect evidence for larval grazing by comparing the epiphytic bacterial composition of two aquatic plants of phytoremediation importance to larval mosquito gut communities.

## Materials and Methods

### Experimental mesocosms and sampling

Five young bulrush seedlings were transplanted into each of eight simulated wetlands [fiber glass mesocosms; area = 1 m^2^] containing 17 cm (depth) of soil mix (plaster sand mixed with peat moss) at the University of California Riverside Aquatic Research Facility on 21 April 2011 (i.e., four replicate alkali bulrush mesocosms and four replicate California bulrush mesocosms). Water was supplied from an irrigation reservoir and water depth was maintained at 17 cm using float valves. The plants received an ambient ammonium nitrogen level of approximately 0.2 mg/L. Microbiota associated with plants, water, and mosquitoes were sampled monthly from September through November. Mosquito and predator abundance was estimated using three 350 mL “standard dip” samples, taken diagonally across each mesocosm every two weeks in September and October, and once in November, after which the mosquito population declined.

Water temperature was recorded at 0.5 h intervals throughout the study using a water temperature data logger (HOBO Water Temp Pro V1, Onset Computer Co.). The average monthly temperatures of the water were 24.2°C, 19.4°C and 12.8°C, in September, October, and November, respectively.

### Bulrush Leaves

Five leaf disks, each 15 mm diameter (oven-dry weight was ~ 0.05 g), were collected monthly from submerged leaves of alkali bulrush and California bulrush in each of the experimental mesocosms, using a cork borer on sterile Petri plates. The disks were placed in sterile 15-mL centrifuge tubes with sterile water. The “rinsates” (detached biofilm) from the Petri plates were also added to the tubes. The cork borer was rinsed with ethanol and flamed between samples. Samples were transported to the laboratory in a cooler on ice. The tubes immediately were sonicated in ice water using an ultrasonic cleaner (Branson 1510) for 10 min to detach bacterial biofilm from the plant surfaces as described previously [23]. After sonication, plant material was removed from the tubes and oven-dried for weight measurement. The suspension left in the tube was centrifuged at 2900 x g in an Allegra 25 centrifuge (Beckman Coulter) at 4°C for 30 min. The majority of the supernatant was then discarded, and the pellet was resuspended in approximately 3 mL of water.

### Water Column

Two water samples from each mesocosm were collected in sterile 50-mL centrifuge tubes on each of the three sampling days and transported to the laboratory on ice. The samples were then centrifuged at 2900 x g for 30 min at 4°C using an Allegra 25 centrifuge. The majority of the supernatant was again discarded and the pellet resuspended in approximately 3 mL of water.

### Mosquito Larvae

Late (third and fourth) instars of *C* *. tarsalis* were sampled from each mesocosm using 350-mL standard dip samples. Five larvae were selected and euthanized immediately in 95% ethanol in sterile 15 mL centrifuge tubes. Samples were transported on ice to the laboratory. The larvae in the tubes were sonicated in an ultrasonic cleaner bath for 2 min at room temperature to detach any *Bacteria* and biofilm from the exoskeleton of the mosquito larvae. The larvae were then rinsed with phosphate buffered saline (PBS) and allowed to air-dry before DNA extraction.

### DNA Extraction from Leaves and Water Samples

Two replicate 0.75-mL volumes from each mesocosm were transferred to individual 2-mL microcentrifuge tubes. Biological material was then pelleted by centrifugation at 9300 x g for 10 min, followed by removal of the supernatant and resuspension in 0.5 mL of nuclease-free water. DNA extraction was carried out with the Ultraclean Soil DNA kit (MoBio Laboratories, Inc, Carlsbad, CA, USA) with some modification to the standard protocol [25]. Specifically, after suspension in nuclease-free water, samples were poured into the 2-mL bead solution provided by the manufacturer. Samples were homogenized using a vortex with a vortex adapter (Va12G20-24; MoBio Laboratories, Inc, Carlsbad, CA, USA) for 10 min at maximum speed, after which 425-µL of the homogenate was transferred to a new 2-mL tube.

### DNA Extraction from Mosquitoes

The DNeasy Blood & Tissue kit (Qiagen, Valencia, CA) was used to extract DNA from a pool of three mosquito larvae per sample following the manufacturer’s protocol with a single final elution in 200 µL of Buffer AE.

### Polymerase Chain Reaction (PCR)

Following DNA extraction, the hypervariable region 3 (V3) of the 16S rRNA gene was amplified using bacterial primers 341F (5’-CCTACGGGAGGCAGCAG-3’) and 518R (5’-ATTACCGCGGCTGCTGG-3’) [26]. For each sample, two replicate 25 µL reactions were conducted, each containing 1X HF Buffer, 200 µM of each dNTP, 1 µM of each primer, 0.5 U of Phusion DNA polymerase (New England Biolabs, Ipswich, MA, USA) and 2 µL of DNA template (concentration 1.4-14.2 ng/µL). Reactions were run on a MasterCycler Gradient 5331 thermocycler (Eppendorf, Hamburg, Germany) with amplification cycle conditions as described previously [27]. Products from the two replicate amplifications were pooled and 20 µL of the combined PCR product was electrophoresed on 1.5% (wt/vol) agarose gel. PCR products of the expected size (170-190 bp) were excised from the gel and cleaned using the High Pure PCR Product Purification Kit (Roche Applied Science). The cleaned PCR product was then quantified using an ND-1000 spectrophotometer (NanoDrop Technologies, USA) and kept at -20°C for library preparation.

### Illumina Library Preparation and Sequencing

Illumina libraries were generated for each sample using NEXTflex DNA sequencing kits (and protocols) and an identifying NEXTflex DNA bar code with 6-base indices (Bioo Scientific, Inc., Austin, TX). Library quality was checked using the 2100 Bioanalyzer (Agilent Technologies, Palo, Alto, CA, USA) and the molar concentration of each library (n=60) was normalized to 7 nM with 10 mM Tris-HCl (pH 8.5). The resulting libraries were pooled, in equimolar quantities, to create three multiplexed libraries and subjected to 101-base paired-end sequencing on a HiSeq 2000 (Illumina, Inc. San Diego, CA) at the Institute for Integrative Genome Biology, Core Instrument Facility, University of California, Riverside.

### Sequence Analysis, Alignment and Taxonomy Assignment

Analysis of the sequence reads was carried out using (Quantitative Insights Into Microbial Ecology) QIIME [28] version 1.4.0 and AXIOME [29] version 1.6.0 pipelines. Non-small subunit and 18S rRNA gene sequences were excluded by aligning all OTUs against a small subunit model using ssu-align [30]. Chloroplast OTUs were excluded with Metaxa [31]. Clustering of identical (0.97 similarity) sequences to operational taxonomic units (OTUs) was carried out using CD-hit-est (multi-threaded version) [32]. Taxonomy assignment was conducted using the Ribosomal Database Project (RDP-II) [33] via QIIME parallel assign_taxonomy_rdp_py script with a confidence level of 0.8. All sequences and associated sample metadata were submitted to MG-RAST (Metagenome Rapid Annotation Using Subsystems Technology) [34] with accession number 4984.

### Statistical analysis

Alpha diversity indices were estimated using QIIME v 1.4.0 and the Phylogenetic Diversity (PD) Whole Tree method for bacterial communities sampled from mosquitoes and each of the two aquatic plants. For analyses which compare between samples, all samples were rarefied down to the smallest library size (9,104 sequences). Differences between OTUs of bacterial communities from three bacterial DNA sources (mosquito larvae, water and plants) were assessed by principal coordinate analysis (PCoA) using an R script [29,35,36]. Beta diversity measures based on both Bray-Curtis dissimilarities of sample OTU profiles and UniFrac distances assessed differences between samples from each of the DNA sources (mosquito, plant and water) and sampling dates. Indicator species analysis was carried out using the R package [37] to determine OTUs that were significantly associated with each of the three sampled habitats. Venn diagrams were also generated to demonstrate OTUs common to both mosquitoes and plant/water habitats. Repeated-measures ANOVA on mosquito and predator abundance, data summary and tables of the bacteria sequences were generated using JMP version 10 [38]. Numbers of mosquito larvae and odonate naiads were transformed by log_10_ (x+1) prior to analysis.

## Results

### Mosquito and Predator abundance

**Figure 1:**
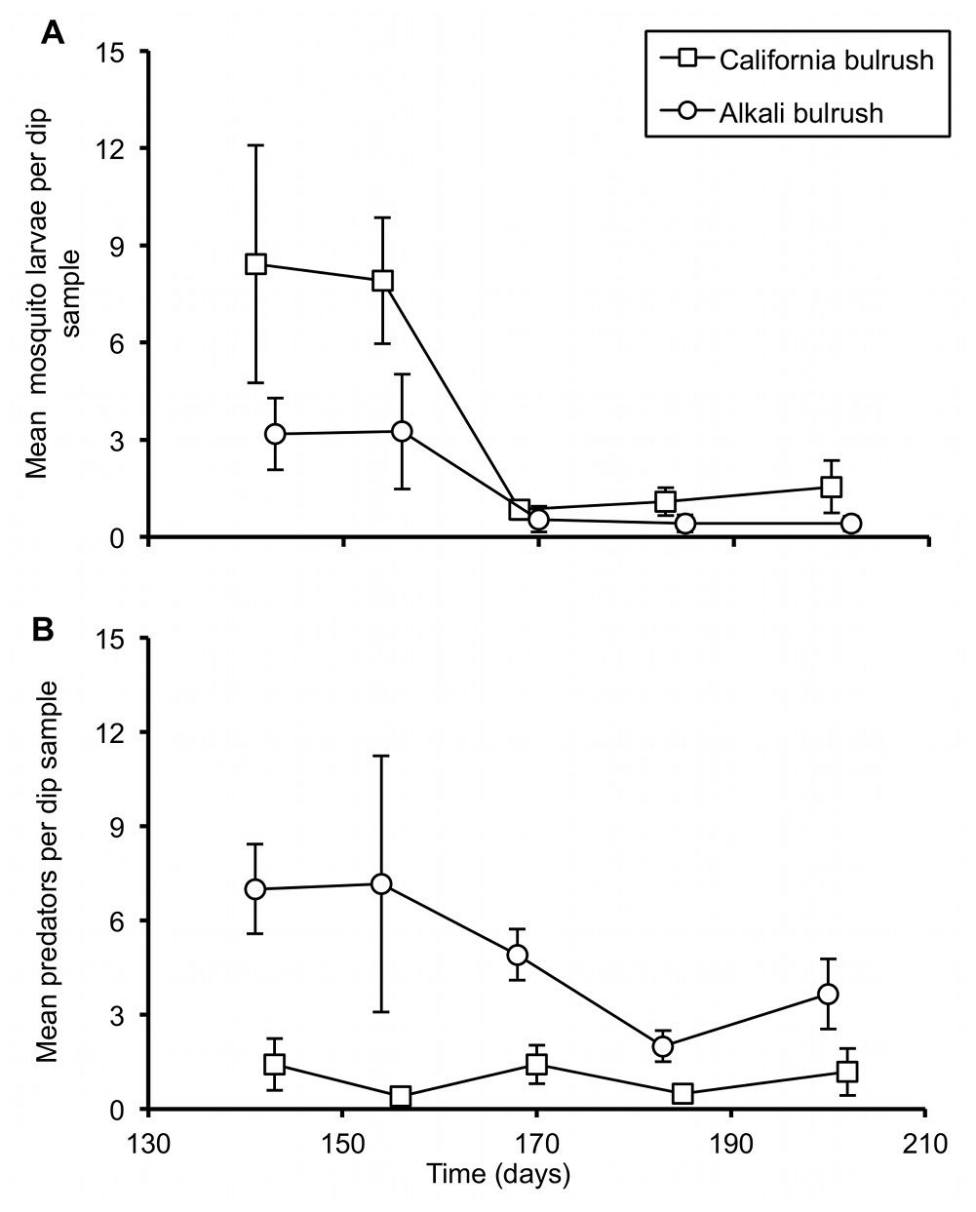
Mosquito and invertebrate predator abundance. Repeated-measures analysis of variance showed that mean numbers of mosquitoes, Panel A and invertebrate predators (zygopteran predators), Panel B varied significantly (p< 0.05) between mesocosms planted with alkali bulrush and California bulrush. Alkali bulrush significantly harbored more predators and fewer mosquitoes as compared to the California bulrush. The x-axis represented time after the onset of the experiment. Error bars represent the standard error of the mean based on four replicate mesocosms per plant species.

Significantly greater (>2-fold) numbers of mosquitoes were observed in mesocosms planted with California bulrush than alkali bulrush (*F*_1,17_= 8.24, *p* < 0.01; Figure 1A). In contrast, mesocosms planted with alkali bulrush produced significantly greater numbers of invertebrate predators (predominantly damselflies, Zygoptera) than did mesocosms containing California bulrush (*F*_1,22_= 36.8, *p* < 0.001; Figure 1B).

### Data Summary of HiSeq2000 Illumina Sequences

We generated a total of 135,838,727 sequences from 60 samples. A large proportion of OTUs identical to eukaryotic 18S rRNA genes (7.0%) and plant chloroplast 16S rRNA genes (1.7%), and all OTUs that did not align to any small subunit model (84.2%) were discarded. A total of 12,177,876 sequences aligned to the bacterial small subunit models and were used in the analysis, which resulted in a total of 123,814 bacterial OTUs. The number of contributed sequences ranged between 9,104–1,336,522 reads per sample.

### Bacteria Diversity and Associations

**Figure 2:**
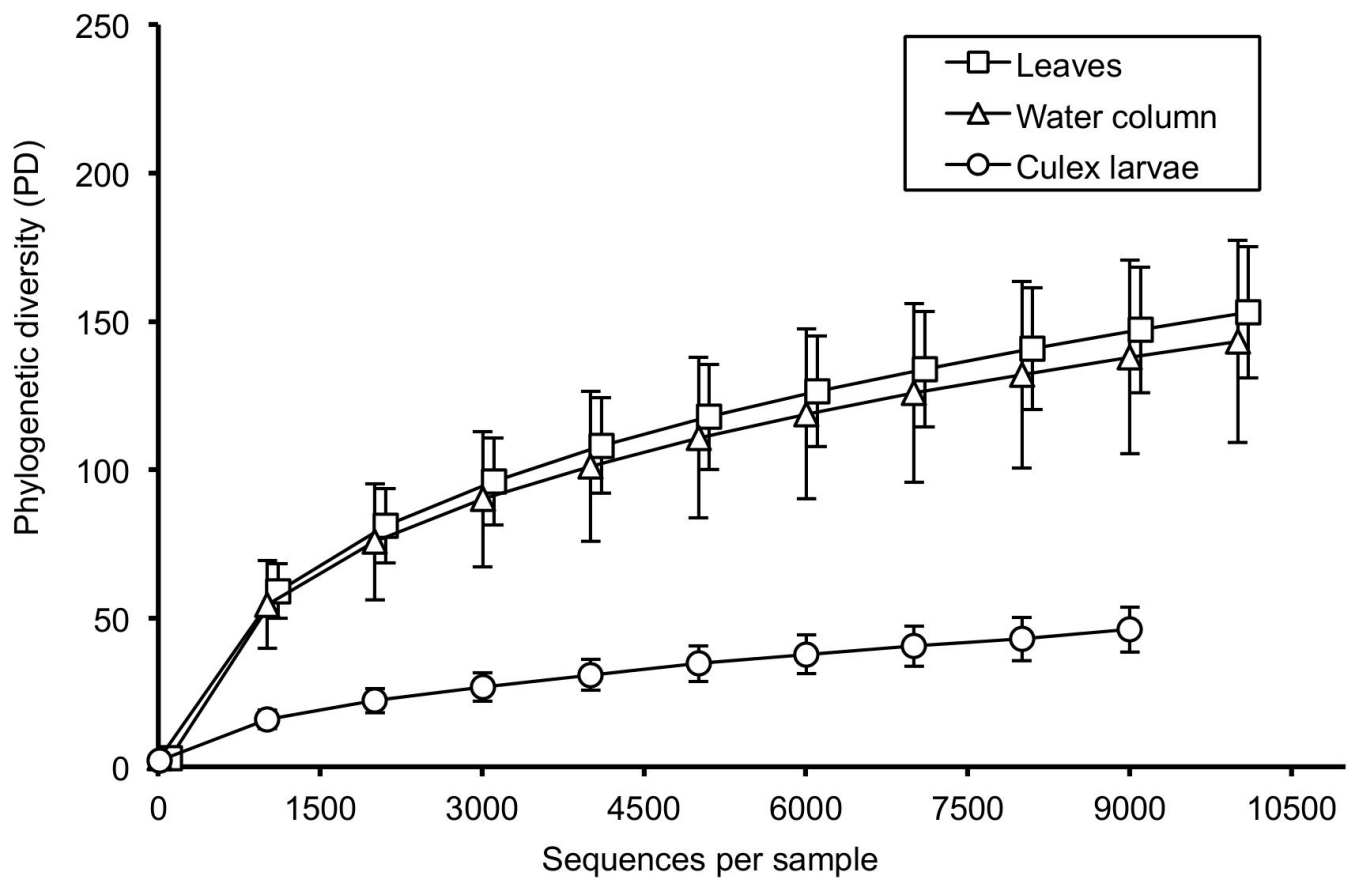
Alpha diversity measures. Alpha diversity measures based on PD_Whole tree of the bacterial communities from mosquito larvae, water column and leaves. Sequences from mosquito samples are significantly less diverse than sequences from water and plant samples. The x-axis for the phylogenetic diversity of *Bacteria* communities from leaf samples is offset by 100 sequences for better illustration.

Alpha diversity based on the PD_Whole tree method revealed that bacterial communities from mosquito samples were significantly less diverse than communities derived from leaf and water samples (Figure 2). There was no significant difference between bacterial communities derived from water column or plant leaves (Figure 2).

**Figure 3:**
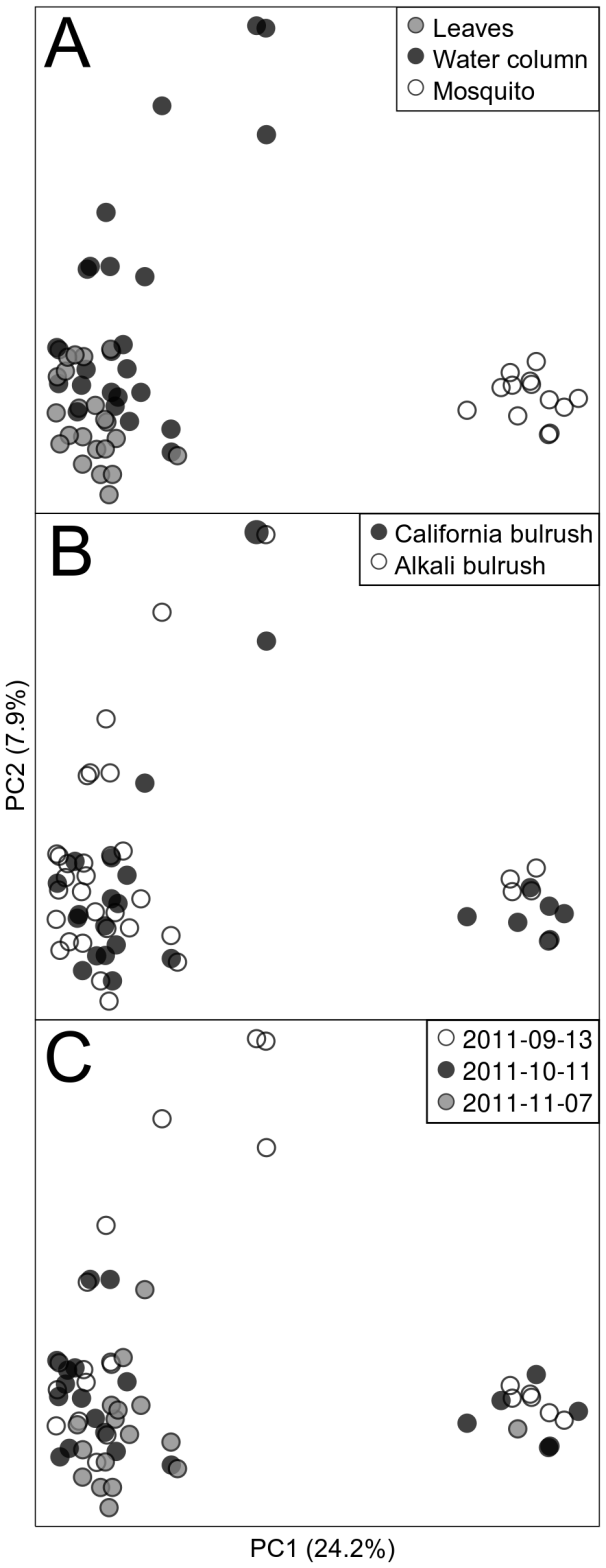
Community similarity of OTU profiles representation. PCoA plots based on Bray-Curtis distances of OTU profiles mosquitoes, water and leaf samples from mesocosms containing the two bulrushes from the different sampling dates. Panel A shows points colored by DNA source. Panel B shows points colored by the plant present in the mesocosm. Panel C shows points colored by sampling date.

**Figure 4:**
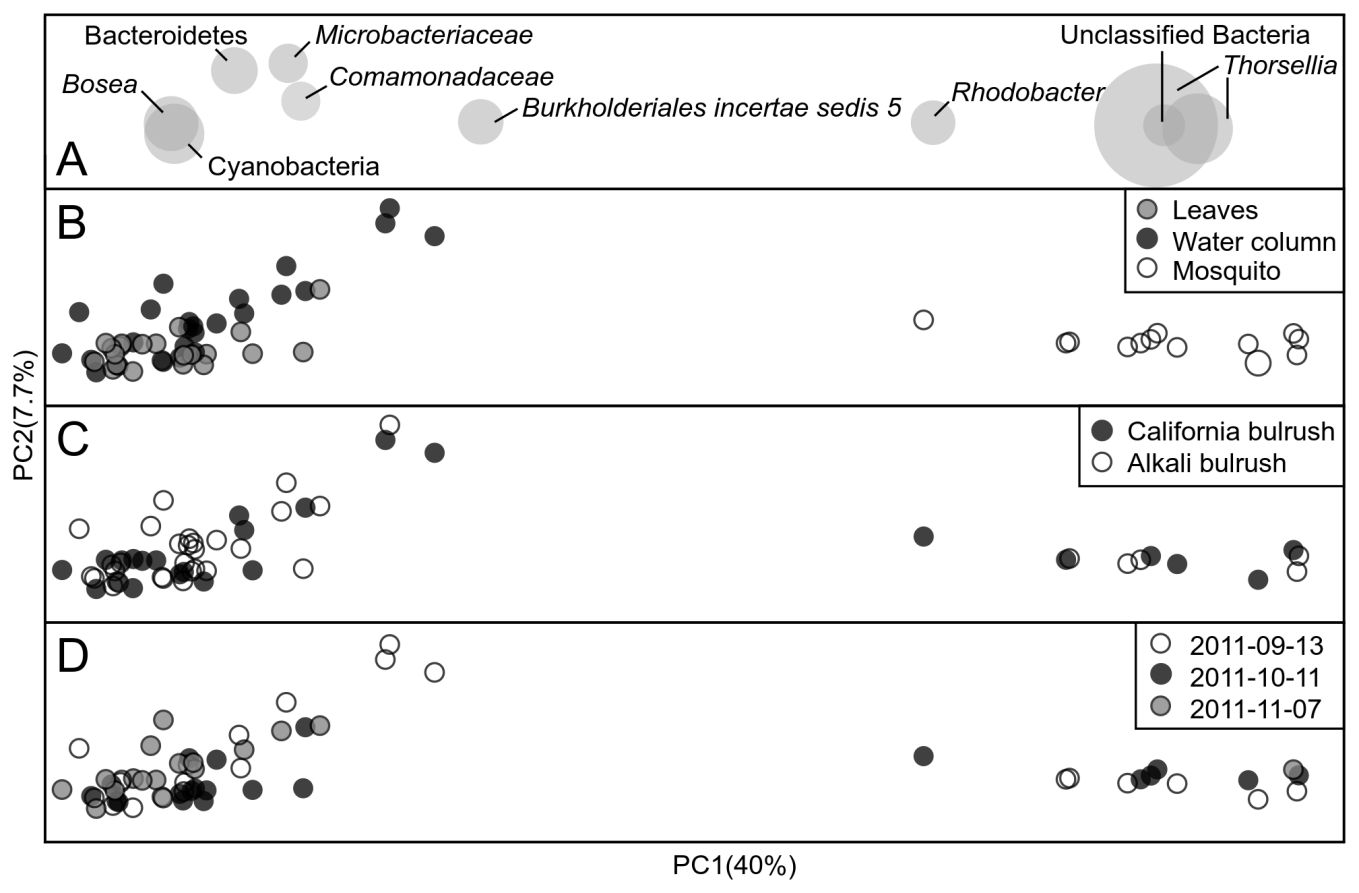
Taxonomic profiling of mosquito-water-plant microbiome profiles. PCoA plots of weighted UniFrac distances of bacterial communities in mosquitoes, water and leaf samples from mesocosms containing the two bulrushes (alkali and California bulrushes) from the three sampling dates. Panel A shows the OTUs associated with that region on the plot, scaled based on sequence abundance. Panel B shows a PCoA plot based on three DNA sources (mosquitoes, water and plant leaves), Panel C recolors samples of Panel B to highlight two plant species, Panel D recolors the same samples based on the three sample dates.

Beta-diversity measures using principal coordinate analysis (PCoA) plots of Bray-Curtis OTU profile distances showed that bacterial communities from the two bulrush species did not differ significantly (Figure 3). Axis 1 of the Bray-Curtis PCoA represented 24.2% of the total variation of the bacterial communities derived from mosquitoes and the two (water and leaf) habitat components. Clustering of samples based on bacterial communities of water and on leaves were primarily influenced by *Cyanobacteria* (OTU #243), Actinobacteria (*Actinobacteriadae*: *Microbacteriaceae* OTU#969), Bacteroidetes (OTU #1002) and Proteobacteria (*Beta*- and *Alphaproteobacteria*; Figure 4). Among the *Betaproteobacteria* subdivision, a member of *Comamondaceae* (OTU #23) and an *Incertaesedis* 5 (*Burkholderiales*; OTU #30) dominated the water and leaf samples. From the *Alphaproteobacteria*, *Bo sea* (OTU #10) dominated the water and leaf samples. Similar to the Bray-Curtis based plot (Figure 3), bacterial communities of mosquito larvae were clustered distinctly from those derived from water and leaves based on weighted UniFrac distances (Figure 4).

**Figure 5:**
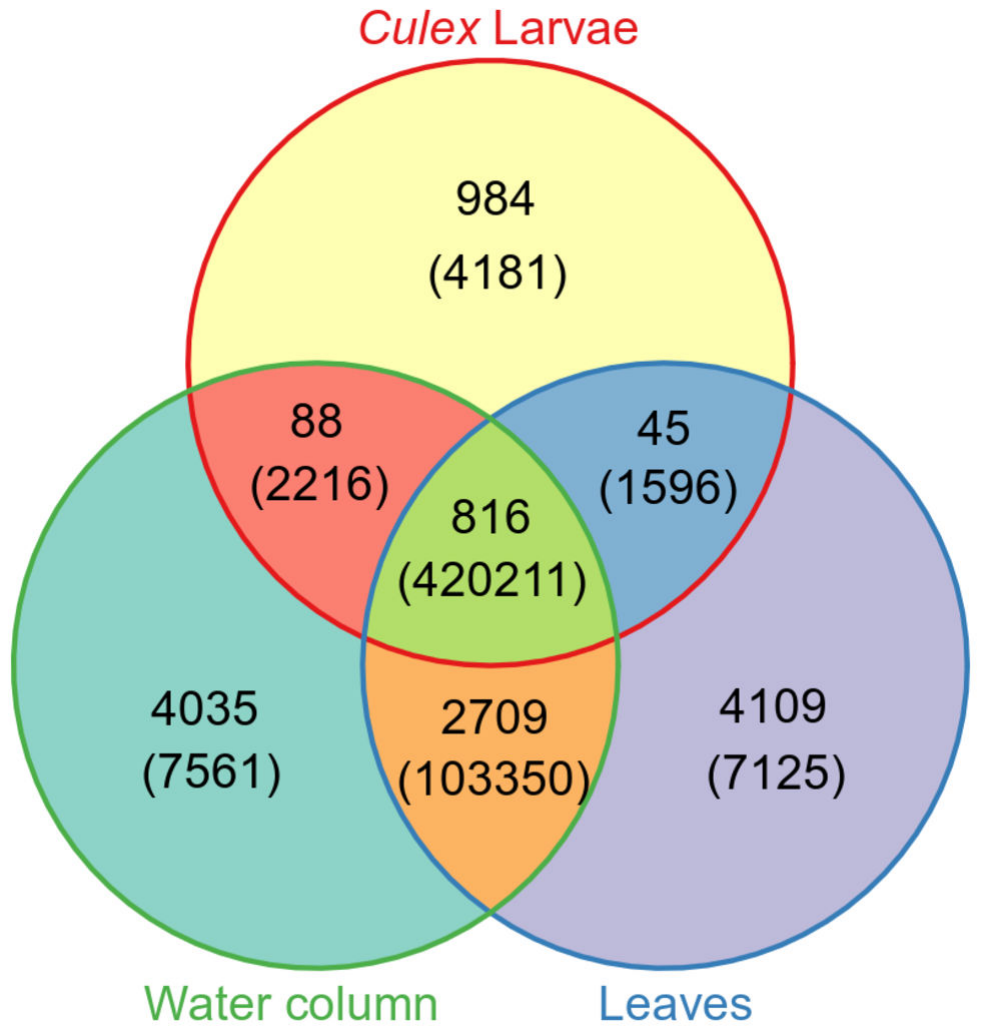
Overlap of bacterial communities across habitats. Venn diagram illustrating overlapping of *Bacteria* OTUs and sequences between mosquito larvae and habitat ( *C* *. tarsalis* larvae; habitat = leaves of alkali and California bulrushes; water = water column samples). The first number represents the number of OTUs, while the number in parentheses represents the number of sequences.

The sequences of 16S rRNA genes of *Bacteria* from both the mosquito gut and shared plant and water habitats showed that a relatively high proportion (49%) of bacterial OTUs in the habitat was found in the guts (Figure 5). Mosquito larvae shared 42% (816 bacterial OTUs) of the bacterial communities with the water column and bulrush leaves (Figure 5) and these shared OTUs accounted for 99% of all sequences recovered from the larvae.

### Bacterial Communities Associated with the Gut of C. tarsalis Larvae

The thirteen larval mosquito samples yielded a total of 1,498,438 bacterial 16S rRNA gene sequences that were assigned to 12,640 OTUs and analyzed to describe the bacterial community of *C* *. tarsalis* larval guts. Of these, 9,514 bacterial OTUs were unique to mosquitoes and were not recovered from other plant and water samples (Figure 5). Among these mosquito-specific OTUs, 22% (2,669 OTUs) were unclassified singletons. Overall, the bacterial communities recovered from the larval guts were classified into 20 phyla and these accounted for12% of the total sequences (Table 1).

**Table 1:** Phylum-level classification of bacterial communities from *C* *. tarsalis* larvae.

Phylum	No. of OTUs	Relative abundance
Proteobacteria	6185	0.6568
Firmicutes	1908	0.2055
Unclassified bacteria	3614	0.0722
Bacteroidetes	479	0.0464
*Cyanobacteria*	196	0.0159
Actinobacteria	196	0.0028
Chloroflexi	6	0.0002
Others*	56	0.0002

* Other bacterial phyla include (Chlamydiae, Acidobacteria, TM7, Gemmatimonadetes, Spirochaetes, Fusobacteria, *Nitrospira* , Chlorobi, Verrucomicrobia, *Deinococcus* - *Thermus* , SR1, WS3, and OP 10)

**Figure 6:**
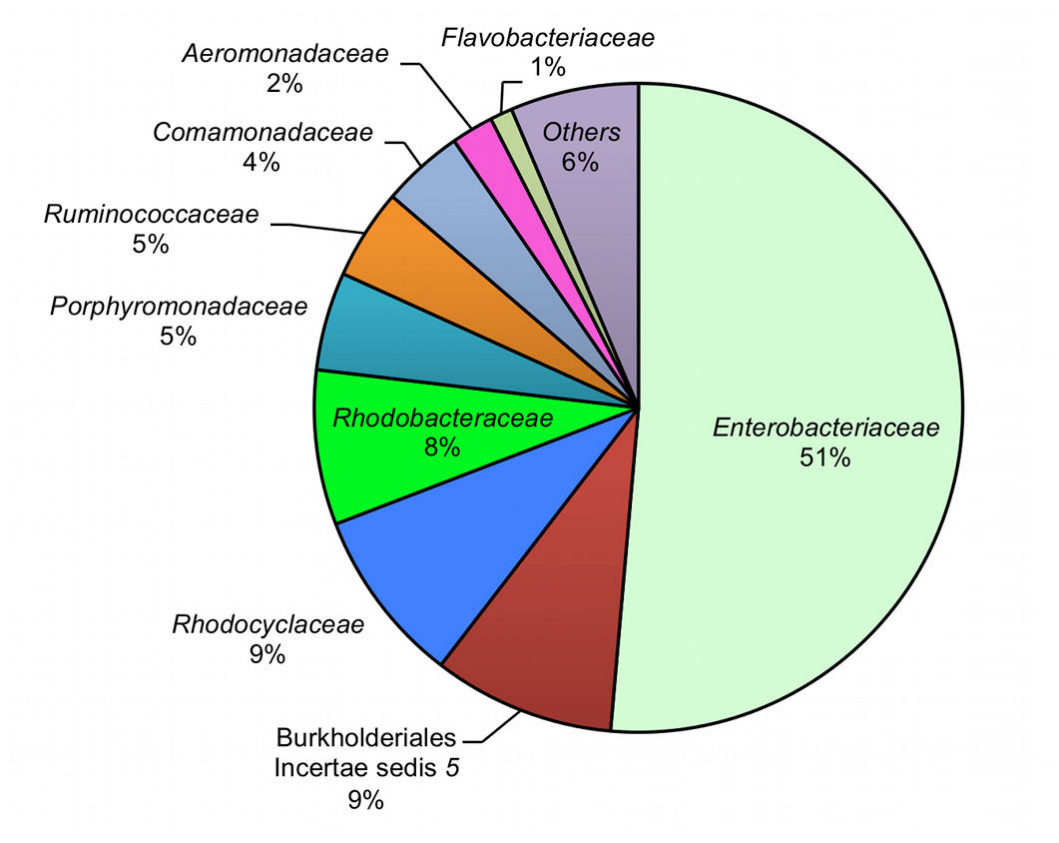
Family-level classification of bacterial communities in mosquitoes. Family-level classification of bacterial communities in *C* *. tarsalis* larvae and their relative proportions.

Proteobacteria dominated the guts of mosquito larvae (Table 1) with three abundant subdivisions: *Gammaproteobacteria* (57%), *Betaproteobacteria* (24%) and *Alphaproteobacteria* (13%). Other members of the Proteobacteria accounted for the remaining 6%. Of the *Gammaproteobacteria*, the family *Enterobacteriaceae* accounted for about 51% of all the larval sequences. The next most common family, *Burkholderiales iIncertae sedis* 5 (*Betaproteobacteria*), accounted for 9% of all the sequences identified to the family level (Figure 6). Overall, 108 families of *Bacteria* were recovered from the guts of *C* *. tarsalis* larvae. Firmicutes, the second most abundant phylum, was dominated by *Clostridiales* (79%).

A total of 738 OTUs of *Thorsellia* (*Gammaproteobacteria*: *Enterobacteriaceae*) were recovered from larval guts, with just three OTUs accounting for 99.5% of *Thorsellia* sequences. Moreover, among the most abundant OTUs (>100 sequences) that were classified to the genus level, 64% were identified to the genus *Thorsellia* (Table 2). Overall, ~250 genera were affiliated with the gut profiles of *C* *. tarsalis* . Amongst our samples, 7 genera were unique to the mosquito gut and the remaining 244 genera were also recovered from the environment (water and leaves). Overall, the larval mosquito gut maintained a fairly stable microbial community regardless of the differences in sampling date and habitats (Figure 4).

**Table 2:** Most abundant (> 100 sequences) genera of *Bacteria* found in *C* *. tarsalis* *larvae*.

Phylum	Class	Family	Genus	a	No. of OTUs
Bacteroidetes	*Bacteroidetes*	*Porphyromonadaceae*	*Dysgonomonas*	6.0	1
	*Flavobacteria*	*Cryomorphaceae*	*Fluviicola*	0.2	1
		*Flavobacteriaceae*	*Flavobacterium*	1.1	1
	*Sphingobacteria*	*Flexibacteraceae*	*Flectobacillus*	0.3	1
Firmicutes	*Clostridia*	*Lachnospiraceae*	*Lachnospiraceae*		
			*Incertaesedis*	0.4	1
Proteobacteria	*Alphaproteobacteria*	*Bradyrhizobiaceae*	*Bo sea*	1.1	2
		*Rhizobiaceae*	*Rhizobium*	0.2	1
		*Rhodobacteraceae*	*Pseudorhodobacter*	0.2	1
			*Rhodobacter*	9.1	2
			*Rubrimonas*	1.0	1
		*Sphingomonadaceae*	*Erythrobacter*	0.3	1
	*Betaproteobacteria*	*Comamonadaceae*	*Hydrogenophaga*	0.9	3
		*Incertaesedis* * 5*	*Leptothrix*	0.2	1
			*Rubrivivax*	0.5	1
		*Oxalobacteraceae*	*Duganella*	0.1	1
		*Rhodocyclaceae*	*Azonexus*	1.9	1
			*Azovibrio*	8.9	2
	*Gammaproteobacteria*	*Aeromonadaceae*	*Aeromonas*	2.6	1
		*Halothiobacillaceae*	*Thiovirga*	1.2	1
		*Enterobacteriaceae*	*Thorsellia*	63.6	6
		*Pseudomonadaceae*	*Pseudomonas*	0.2	4

a relative percentages (only percentages ≥ 0.1% are shown).

### Dominant Bacterial Communities Associated with Habitats

A total of 22 bacterial phyla were found in water samples whereas 23 phyla were recovered from the bulrushes. A small number of Planctomycetes was detected in the plant samples, but not found in the water samples. Proteobacteria, Bacteroidetes, Actinobacteria and *Cyanobacteria* were the most abundant phyla found in the water column and on the submerged leaves of alkali and California bulrushes (Table S1). These four phyla accounted for 84% of all sequences from water column and unclassified bacterial OTUs accounted nearly 15% of water column sequences (Table S1). Plant and water samples were specifically clustered with *Comamonadaceae*, *Microbacteriaceae*, *Bo sea*, *Burkholderiales* and *Cyanobacteria* species (Figure 4).

### Indicator Species

Analysis of indicator species [39] of bacterial OTUs from mosquito (Table 3) and plant/water (Table 4) samples revealed OTUs that strongly associated with one group over the others. The indicator species concept has widely been applied in vegetation ecology studies over the last two decades [39] to typify habitats or groups by taking in to account the abundance and frequency of a species that occur in these habitats or groups. A maximum indicator species value of 1.0 represents the species occurrences in all samples of a treatment group (fidelity) and only in samples from that treatment group (specificity); lower indicator values indicate that OTUs are not good predictors of treatment groups or habitats [39]. There were 99 bacterial OTUs identified as indicator species from mosquito larvae (*p* < 0.05), of which 81 were classified to taxa below phyla level (Table S2). The remaining 18 indicator OTUs were unclassified. Of the identified indicator OTUs, the genus *Thorsellia* (19 OTUs) was the dominant predictor of the *Bacteria* community from *Culex* larvae. Members of Proteobacteria (71%, by sequence abundance), Firmicutes (20%), Bacteroidetes (3%) were also among the dominant predictors of bacterial communities from the gut of *Culex* larvae. In addition, among the OTUs that had indicator values greater than 0.8 (31 OTUs), 63% of these bacterial OTUs (by sequence abundance) were originated from mosquito larvae whereas water and the bulrushes had only 25% and 12%, respectively, suggesting that there was consistency within bacterial communities associated with mosquito larvae (Table S2).

**Table 3:** Indicator species of bacterial OTUs from the guts of *Culex* mosquito cluster (*p* < 0.05).

OTU ID	No. of sequences	Consensus Lineage	Ind Val. ^a^
3	303441	Proteobacteria; *Gammaproteobacteria*; *Enterobacteriales*; *Enterobacteriaceae*; *Thorsellia*	0.998
25	216058	Proteobacteria; *Gammaproteobacteria*; *Enterobacteriales*; *Enterobacteriaceae*; *Thorsellia*	0.998
272	52381	Proteobacteria; *Betaproteobacteria*; *Rhodocyclales*; *Rhodocyclaceae*; *Azovibrio*	0.99
4	80284	Proteobacteria; *Alphaproteobacteria*; *Rhodobacterales*; *Rhodobacteraceae*; *Rhodobacter*	0.98
528	4212	Proteobacteria	0.92
82	56986	Firmicutes; "*Clostridia*"; *Clostridiales*	0.92
5	49398	Bacteroidetes; *Bacteroidetes*; *Bacteroidales*; *Porphyromonadaceae*; *Dysgonomonas*	0.92
27	44364	Firmicutes	0.92
384	20555	Firmicutes; "*Clostridia*"; *Clostridiales*	0.92
30	127850	Proteobacteria; *Betaproteobacteria*; *Burkholderiales*; Incertae sedis 5	0.88
1284	431	Proteobacteria; *Gammaproteobacteria*; *Enterobacteriales*; *Enterobacteriaceae*; *Thorsellia*	0.85
31	44685	Firmicutes; "*Clostridia*"; *Clostridiales*; "*Ruminococcaceae*"	0.84
12	75489	Firmicutes; "*Clostridia*"; *Clostridiales*	0.84

a Only OTUs with indicator values > 0.8 and identified to phylum level and below are shown.

The entire indicator value table for all bacterial OTUs from this study included in the supplementary material.

**Table 4:** Top indicator species values for bacterial OTUs from water column and bulrush leaves (*p* < 0.05).

OTU ID	No. of sequences	Consensus Lineage				Cluster	Ind Val. ^a^
10551	27680	Proteobacteria; *Alphaproteobacteria*; *Rhodospirillales*				plant	0.90
8931	4490	Proteobacteria; *Betaproteobacteria*; *Burkholderiales*				plant	0.89
3058	14532	Proteobacteria; *Alphaproteobacteria*; *Rhizobiales*; *Hyphomicrobiaceae*; *Devosia*				plant	0.89
10374	10421	Proteobacteria; *Betaproteobacteria*; *Burkholderiales*				plant	0.88
3595	30290	Proteobacteria; *Alphaproteobacteria*; *Rhizobiales*				plant	0.88
16356	4868	Proteobacteria; *Alphaproteobacteria*				plant	0.87
8991	39373	Bacteroidetes; *Flavobacteria*; *Flavobacteriales*; *Flavobacteriaceae*; *Mariniflexile*				plant	0.85
8986	4399	Bacteroidetes; *Sphingobacteria*; *Sphingobacteriales*				plant	0.84
1252	7869	Bacteroidetes				plant	0.84
10964	16015	Proteobacteria; *Betaproteobacteria*; *Rhodocyclales*; *Rhodocyclaceae*; *Zoogloea*				plant	0.83
9696	10452	Bacteroidetes; *Flavobacteria*; *Flavobacteriales*; *Flavobacteriaceae*				plant	0.83
22257	4776	Bacteroidetes; *Sphingobacteria*; *Sphingobacteriales*; *Flexibacteraceae*; *Runella*				plant	0.83
4395	3113	Bacteroidetes; *Sphingobacteria*; *Sphingobacteriales*; *Flexibacteraceae*; *Dyadobacter*				plant	0.82
2649	43461	Proteobacteria; *Alphaproteobacteria*; *Sphingomonadales*; *Sphingomonadaceae*; *Sphingobium*				plant	0.80
969	151929	Actinobacteria; *Actinobacteria*; *Actinobacteridae*; *Actinomycetales*; *Micrococcineae; Microbacteriaceae*				water	0.87
3802	67333	Proteobacteria; *Betaproteobacteria*; *Burkholderiales*				water	0.86
1002	229663	Bacteroidetes				water	0.82

a Only OTUs with indicator values > 0.8 and identified to phylum level and below are shown.

Members of the Actinobacteria and Proteobacteria phyla sequenced from water samples had the highest indicator values, whereas several members of Proteobacteria and Bacteroidetes were good predictors of the bacterial communities associated with bulrush leaves (Table 4). *Alphaproteobacteria* specifically dominated the bulrushes while *Betaproteobacteria* dominated the water column.

## Discussion

This study represents the first use of next-generation sequencing to explore the bacterial communities associated with the guts of mosquito larvae and associated habitats (water column and emergent macrophytes). Although it has been suggested that immature mosquitoes feed on microbial assemblages [5], existing evidence supporting this hypothesis is mostly limited to characterization of microorganisms based on traditional cultivation, morphology and Sanger sequencing techniques [8,9,40,41]. These methodologies undoubtedly under-sampled the diversity of microflora found in mosquito guts and the environment.

Although the majority of ingested microbes are likely to be quickly digested and/or passed through the gut, at the time of sampling, almost half (49%) of the bacterial OTUs (representing 99% of sequences) associated with plant and aquatic samples were found in the mosquito gut (Figure 5). *Culex* species are not known to be inherently selective feeders, however, their gut might be selective and only provide suitable medium for the proliferation of a few *Bacteria*. Three OTUs of the most abundant genus, *Thorsellia* , were among those recovered both from larval guts, water and plant samples. All the phyla of *Bacteria* originated from mosquitoes were represented in the water column and on leaves.

Kaufman and colleagues compared the bacterial communities found in treeholes inhabited by mosquitoes, to treeholes that lacked mosquitoes and found differences in the bacterial communities of the two habitats [41]. However, no effort was made to eliminate mosquitoes from pond mesocosms and make a comparison of bacterial communities in the presence and absence of mosquitoes in this study. To circumvent this limitation, we created simulated wetlands planted with two bulrush species that influenced the abundance of mosquitoes and their predators (Figure 1). It is well known that aquatic plants of different structural complexities have a differential effect on macroinvertebrate community assemblages (e.g., [23]). Alkali and California bulrushes have different structural complexities that likely influence the presence and abundance of mosquitoes and their predators (odonate naiads). Alkali bulrush has a more structurally complex growth habit with significantly greater number (5.8 ± 0.23 SE) of leaves than California bulrush (2.2 ± 0.12 SE). However, the bacterial communities in the guts of larval mosquitoes from mesocosms planted with the two bulrushes species did not differ significantly between monotypic plots of the two bulrushes (Figure 3). Our results indicated that differences in the abundance of mosquitoes and mosquito-predators did not affect the diversity of the microbial community in the two bulrushes, according to the top-down predation model. However, we did not examine other bacterivores (e.g., protists), the presence or absence of which might have also affected bacterial community composition.

Similar to our findings, a previous study did not observe changes in bacterial communities in two species of syrphid flies that fed on different larval resources and concluded that the insect midgut might be selective for certain species of microbes [42]. That study also found *Enterobacteriaceae* (different genera from what were found in our study) were the predominant colonizers of larval guts, suggesting that the gut of insects might be conducive for the proliferation of this group of *Bacteria*. Additional studies of dipteran larvae showed that the bacterial communities in *Drosophila* spp. and *Musca* *domestica* were dominated by several members of *Enterobacteriaceae* [43,44].

This study identified for the first time that *Thorsellia* (Proteobacteria, *Enterobacteriaceae*) OTUs dominated the gut of *C* *. tarsalis* larvae collected from natural habitats (Figure 4). *Thorsellia* *anophelis* was first isolated from the midgut of adult *Anopheles* *arabiensis* mosquitoes [45]. It has been suggested that this dominant bacterium may be acquired from rice paddies via ingestion by the larvae, and transferred transstadially from larvae to adults [46]. *Thorsellia* are Gram-negative, rod-shaped bacteria [47] and have recently been suggested for manipulation of mosquitoes that transmit malaria parasites [48]. Although the current taxonomical category using RDP and NBCI databases places *Thorsellia* in the *Enterobacteriaceae* family, the correct taxonomical assignment of this genus within *Gammaproteobacteria* is still unresolved [47,48].

The *Thorsellia* genus has never been reported from mosquitoes outside *Anopheles* and this was the first report of their presence in *Culex* mosquitoes. Of all the *Thorsellia* sequences obtained in this study, 99.6% were from *C* *. tarsalis* larvae; leaf samples and water column contained only ~0.2% and ~0.3% of *Thorsellia* sequences, respectively. This suggests that the genus is a well-established mosquito gut inhabitant, although the habitat may still serve as a reservoir [48].

It is currently unknown whether members of *Thorsellia* are obligate symbionts of mosquitoes but their close association with the gut of the mosquito might indicate that this genus is at least a commensal that can be acquired from the habitat reservoir and proliferates in the insect midgut. It is evident from our study and [48] that this genus is likely free living because it was recovered from the environment. The fact that this genus is found in both *Culex* and *Anopheles* supports it as a potential candidate for manipulating disease vectors across genera. Midgut bacteria have been known to prime the native immunity of *Anopheles* [49,50] and whether the genus *Thorsellia* has the capacity to make mosquitoes refractory to parasites and could be used for the symbiotic control strategy in *Culex* genera has yet to be explored.

The bacterial communities dominating the water column included Actinobacteria, Bacteroidetes, *Cyanobacteria* , *Betaproteobacteria*, and other phyla typically found in freshwater habitats [51,52]. The submerged plant leaves were dominantly colonized by *Alphaproteobacteria*, *Betaproteobacteria*, *Gammaproteobacteria*, *Cyanobacteria* and *Sphingobacteria* also typically found on plant leaves [24,52]. Similar to our findings, Tanaka and coworkers also found the family *Comamonadaceae* (*Betaproteobacteria*) to be the most abundant family predominating in pond water [52]. However, the roots of common reeds, an aquatic plant commonly planted for bioremediation importance, contained a separate group of *Betaproteobacteria* from the pond water indicative of the plant rhizosphere having an influence on the surrounding microbial communities [20,52]. In our study, we did not find significant separation of the bacterial communities associated with submerged portion of plants from those found in the water column (Figure 3). We also did not characterize the microbial communities associated with the roots of the two bulrushes, which might be an important area of future study and significant in understanding their role in treatment wetlands [52]. In this study, we also characterized for the first time the bacterial communities associated with the submerged portions of two bulrushes that are used in wastewater treatment processes.

Next-generation technologies, such as Illumina, provide unprecedented access to environmental bacterial communities, including those from the guts of disease vectoring insects [2,27,53]. Future research will investigate the role of these dominant groups of *Bacteria* identified in this study across the developmental stages of *C* *. tarsalis* . Future studies will also investigate whether congeneric *Culex* species that share common niches also share similar gut microbiota*.*

## Supporting Information

Table S1 Major phyla of bacterial OTUs associated with the habitat of *Culex* larvae.
(DOCX)

Table S2 Indicator species (p<0.05) based on bacterial DNA sampled from mosquito larvae, bulrush leaves and water column.
(XLS)
