# Supplementary material for: Bacterial Communities Associated with Culex Mosquito Larvae and Two Emergent Aquatic Plants of Bioremediation Importance
Source: PLoS One. 2013 Aug 15;8(8):e72522. doi: 10.1371/journal.pone.0072522 (PMC3744470; doi:10.1371/journal.pone.0072522)
Supplement: Table S1 — Major phyla of bacterial OTUs associated with the habitat of Culex larvae. (DOCX) [file pone.0072522.s001.docx]

Table S1. Major phyla of bacterial OTUs associated with the habitat of *Culex* larvae.

| Phylum | Number of OTUs | | Relative proportion | |
| --- | --- | --- | --- | --- |
|  | water | bulrush | water | bulrush |
| Acidobacteria | 144 | 238 | 0.0030 | 0.0064 |
| Actinobacteria | 1910 | 993 | 0.0505 | 0.0095 |
| Aquificae | 1 | 1 | <0.0001 | <0.0001 |
| BRC1 | 16 | 13 | <0.0001 | <0.0001 |
| Bacteroidetes | 4557 | 4750 | 0.1796 | 0.1426 |
| Chlamydiae | 32 | 31 | 0.0001 | 0.0002 |
| Chlorobi | 2 | 2 | 0.0001 | <0.0001 |
| Chloroflexi | 58 | 76 | 0.0005 | 0.0007 |
| Cyanobacteria | 1793 | 2726 | 0.0624 | 0.0902 |
| Deinococcus-Thermus | 3 | 6 | <0.0001 | <0.0001 |
| Firmicutes | 926 | 912 | 0.0078 | 0.0043 |
| Fusobacteria | 8 | 8 | 0.0001 | <0.0001 |
| Gemmatimonadetes | 35 | 38 | 0.0014 | 0.0033 |
| Nitrospira | 11 | 8 | 0.0001 | <0.0001 |
| Unclassified | 18963 | 22643 | 0.1504 | 0.1540 |
| OP10 | 8 | 16 | 0.0001 | 0.0004 |
| Planctomycetes | - | 1 | - | <0.0001 |
| Proteobacteria | 30879 | 38346 | 0.5425 | 0.5862 |
| SR1 | 7 | 5 | 0.0002 | 0.0002 |
| Spirochaetes | 43 | 49 | 0.0009 | 0.0010 |
| TM7 | 16 | 31 | 0.0002 | 0.0009 |
| Tenericutes | 2 | 1 | <0.0001 | <0.0001 |
| Verrucomicrobia | 15 | 15 | <0.0001 | <0.0001 |
| WS3 | 7 | 5 | 0.0002 | 0.0001 |
